# Botulinum Neurotoxins: Qualitative and Quantitative Analysis Using the Mouse Phrenic Nerve Hemidiaphragm Assay (MPN)

**DOI:** 10.3390/toxins7124855

**Published:** 2015-11-25

**Authors:** Hans Bigalke, Andreas Rummel

**Affiliations:** toxogen GmbH, Feodor-Lynen-Strasse 35, 30625 Hannover, Germany; rummel@toxogen.de

**Keywords:** mouse phrenic nerve hemidiaphragm assay, botulinum neurotoxin, detection, quantification, animal replacement method, complex matrix

## Abstract

The historical method for the detection of botulinum neurotoxin (BoNT) is represented by the mouse bioassay (MBA) measuring the animal survival rate. Since the endpoint of the MBA is the death of the mice due to paralysis of the respiratory muscle, an *ex vivo* animal replacement method, called mouse phrenic nerve (MPN) assay, employs the isolated *N. phrenicus*-hemidiaphragm tissue. Here, BoNT causes a dose-dependent characteristic decrease of the contraction amplitude of the indirectly stimulated muscle. Within the EQuATox BoNT proficiency 13 test samples were analysed using the MPN assay by serial dilution to a bath concentration resulting in a paralysis time within the range of calibration curves generated with BoNT/A, B and E standards, respectively. For serotype identification the diluted samples were pre-incubated with polyclonal anti-BoNT/A, B or E antitoxin or a combination of each. All 13 samples were qualitatively correctly identified thereby delivering superior results compared to single *in vitro* methods like LFA, ELISA and LC-MS/MS. Having characterized the BoNT serotype, the final bath concentrations were calculated using the calibration curves and then multiplied by the respective dilution factor to obtain the sample concentration. Depending on the source of the BoNT standards used, the quantitation of ten BoNT/A containing samples delivered a mean *z*-score of 7 and of three BoNT/B or BoNT/E containing samples *z*-scores <2, respectively.

## 1. Introduction

**Figure 1:**
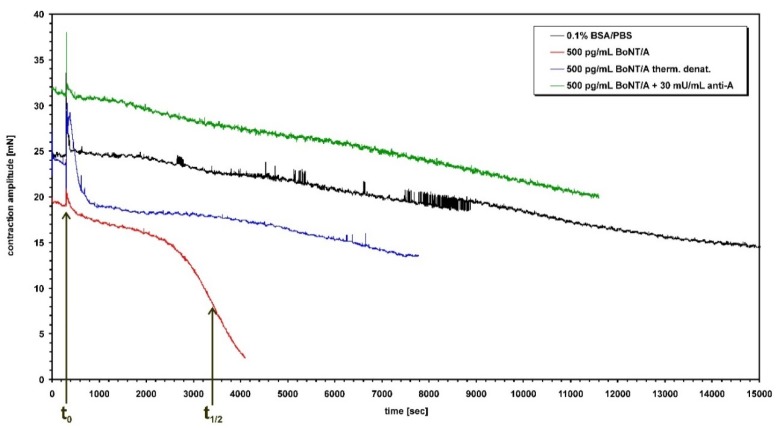
Time course of hemidiaphragm paralysis caused by BoNT/A. The amplitude of muscle contraction in mN represents the difference between recorded basal and maximal tensions. All samples were administered at *t*_0_ = 300 s. Addition of 0.1% BSA/PBS lead to spontaneous reduction of only ~35% over a period of 4 h. Upon administration of 500 pg/mL BoNT/A the amplitude remained unchanged for a dose dependent latent period and then decayed in a steep sigmoidale curve down to zero. The time period between application *t*_0_ and the inflection point when the contraction amplitude halved, the so called paralysis time *t*_1/2_, was used for construction of the calibration curves (Figure 2). Whereas 500 pg/mL BoNT/A yielded *t*_1/2_ = 51 min, thermal denaturation or neutralization by serotype-specific antiserum of BoNT/A did not cause paralysis, but only led to spontaneous partial reduction of the contraction amplitude similar to the negative control sample.

The potential misuse of botulinum neurotoxins (BoNTs) as biological weapons, the accidental intoxication through food and their increasing applications as therapeutic drugs for treatment of many neurological and non-neurological disorders require meaningful methods for detection and quantification. Because of their high potencies, the concentrations of deadly or clinically effective doses are far below the detection limit of most standard chemical detection methods. Furthermore, pure chemical methods do not differentiate between active and inactive BoNT or peptidic fragments thereof [1]. Therefore, the method of first choice is a reliable bioassay that can detect BoNT concentrations in the low nanogram or upper picogram range. For decades, the mouse bioassay (MBA) determining the median lethal dose (MLD) of BoNT in mice represented the gold standard among various biological, chemical or immunological detection systems for BoNT [1,2]. The MLD can easily be calculated from the numbers of deceased *versus* surviving mice after treatment with increasing doses of toxin [3,4,5,6] and has been accepted by pharmaceutical regulatory agencies worldwide as well as being implemented in official standard operating procedures like German DIN10102 [7] and US Association of Official Analytical Chemists (AOAC Official Method of Analysis 977.26) [8] for detection of BoNT in food. It comprises, however, many disadvantages. The MBA is costly, it lasts for a long period (up to 5 days) and, most important, many mice suffer from botulism and die painfully by respiratory failure due to flaccid paralysis of the diaphragm muscles which represents its major end point [9]. The death of mice, however, could also be caused by a wide range of other systemic effects, *i.e.*, pneumonia or heart failure causing limited specificity and precision of the MBA. A shortened version of the MBA is the mouse time-to-death method employing high concentrations of BoNT which reduces the experimental time to a single day [10]. Local paralysis assays like the digital abductions score (DAS) are sublethal and reflect the pharmacokinetics of intra muscularly injected BoNT, but still constitute an animal experiment and lack precision due to subjective read out. In contrast, the isolated mouse phrenic nerve (MPN) hemidiaphragm assay, an *ex vivo* method examining the full physiological pharmacodynamic of BoNT by closely reproducing *in vivo* respiratory failure, replaces the MBA since sacrificing animals, e.g., for scientific purposes, is by definition not an animal experiment. A precursor of the MPN test performed with rat organs was first published by Bülbring in 1946 [11] and later adapted to tissue of mice [12,13]. The change of the species increased the sensitivity of the test dramatically. However, no difference in the paralytic half-time was observed, e.g., when phrenic nerve hemidiaphragm preparations from mice of outbred strain Naval Medical Research Institute (NMRI) and inbred strain C57BL/6 were compared [14]. Furthermore, the MPN assay not only replaces animal experiments, it also reduces consumption of mice. Whereas LD_50_ determination of a single BoNT by MBA requires at least 100 mice (10 BoNT dilutions for groups of 10 mice) [4], the MPN assay requires less than 15 hemidiaphragm preparations. In addition, although only the left phrenic nerve is nicely exposed after opening the chest an experienced operator can also successfully dissect the right phrenic nerve despite being located behind vital organs and closely attached to main blood vessels. Hereby, the use of left and right hemidiaphragms further halves the consumption of animals. After the application of BoNT to an organ bath in which the MPN preparation has been mounted, the contraction amplitude of the indirectly stimulated muscle declined after a short lag time continuously in a characteristic sigmoidale pattern until complete paralysis occurs (Figure 1). The contractions of the hemidiaphragm are recorded via a force transducer and appropriate hard- and software for analysis over time. The time period between application of BoNT into the organ bath and the time point when the contraction amplitude is halved to its original value (paralysis time or *t*_1/2_) is the read out to determine the presence of BoNT, its efficacy and potency as well as its concentration in comparison to BoNT standard material. It has been demonstrated that the paralysis time correlates with the toxicity (MLD, LD_50_, Units) determined by the MBA [15]. Thus, the MPN is precise, reduces consumption of animals, replaces animal experiments, and hence represents a superior substitute for the mouse bioassay [16]. Therefore, it is listed, e.g., as an alternative method for assaying pharmaceutical preparations of injectable BoNT/A in the European Pharmacopoeia [17]. In addition, the MPN assay has been used in numerous scientific publications to decipher the mechanism of action of botulinum neurotoxins, identify its cellular receptors and screen for inhibitors of BoNT [13,14,18,19,20,21,22,23,24,25,26,27,28,29,30,31,32,33,34,35,36]. Furthermore, the MPN assay is suitable to identify BoNT serotypes employing neutralizing antitoxins, characterize neutralizing antibodies as pharmaceutical countermeasure against botulism and measure the occurrence of neutralizing anti-BoNT antibodies in patients’ sera treated with BoNT pharmaceuticals [37,38,39,40,41,42,43,44,45,46,47,48,49,50,51,52]. The MPN has also been proven in the real world. The detection of BoNT/B in ham has helped physicians to find the right diagnosis (BoNT/B poisoning) and appropriate treatment in an unclear human case marked by neurological symptoms [53].

Here, the MPN assay was employed to analyze complex samples delivered within the international EQuATox BoNT proficiency test organized by the Robert Koch-Institut comprising 24 laboratories [54]. All 13 samples spiked with different amounts of in depth characterized recombinant BoNT reference materials [55] (Table 1) were qualitatively correctly identified by the MPN assay. Employing in house BoNT calibration curves the quantitation of ten BoNT/A containing samples delivered a mean *z*-score of 7 and of three BoNT/B or BoNT/E containing samples *z*-scores <2.

## 2. Materials and Methods

### 2.1. Materials

Standards of pure BoNT (150 kDa) were obtained from List Biological Laboratories, USA (BoNT/A) and manufactured recombinantly by toxogen GmbH, Germany, (BoNT/B and E), respectively. Anti-BoNT/A (300 IU/mL), anti-BoNT/B (500 IU/mL) and anti-BoNT/E (estimated 100 IU/mL) antisera (rabbit) were kindly provided by Jürgen Frevert, Battelle Institute, Frankfurt, Germany. Cross reactivity was checked against BoNT/A, B and E at serotype specific neutralizing titers (anti-A 0.75 mU/mL; anti-B 1.25 mU/mL; anti-E 0.25 mU/mL). All antisera lacked cross reactivity against the other two serotypes. The 13 samples to be analyzed were received in liquid form, one mL each and stored at a temperature between 4–8 °C. Aliquots of the samples were directly diluted in Earl’s Balanced Salt Solution (EBSS) and if necessary diluted further stepwise. In case dilution was 1:40 or lower, the samples were dialyzed against EBSS before dilution to prevent effects of matrices caused by differing ion concentrations. If the course of paralysis had shown an untypical trend in this study, the samples would have been dialyzed also at lower dilutions. The equipment and software were purchased from Föhr Medical Instruments GmbH (FMI), Seeheim, Germany.

**Table 1:** Detection and quantification of BoNT in 13 PT samples. Paralysis times were determined based on various dilutions of the samples employing the MPN assay. In the first run, samples were diluted 1:1000 and tested for paralysing activity. Depending on the value of the paralysis time, higher or lower dilutions were tested for activity to obtain paralysis times within the range of the calibration curves (Figure 2). Upon serotype identification (Table 2) the paralysis times within the grey fields were chosen for the calculation of the concentration of BoNT except for sample 8. The individual concentration of BoNT/A and B in sample 8 was calculated employing paralysis times obtained upon complete neutralization of the other serotype and listed in Table 2.

Sample	Serotype	Matrix	Dilution Factor of Sample									BoNT Concentration						*z* (BoNT)	*Q* (BoNT)	(%)
			10,000	2000	1000	500	200	100	50	40	13	X_1_ (ng/mL)	X_2_ (ng/mL)	*N*	X (ng/mL)	X_a_ (ng/mL)	D (ng/mL)			
			Measured Paralysis Time t_1/2_ (min)																	
S1	BoNT/A	Meat extract			142		69/78					44.0	27.0	2	35.5	10.5	25.0	**9.3**	2.39	239
S2	BoNT/A	0.1% BSA/PBS			120		88/83					17.4	21.8	2	19.6	9.9	9.7	**3.9**	0.98	98
S3	**-**	0.1% BSA/PBS			>160		>180			>180	>180			4	**-**	**-**	**-**	-	**-**	**-**
S4	BoNT/E	0.1% BSA/PBS			154		116	118	92/82			12.5	18.3	2	15.4	10.9	4.5	**1.6**	0.42	42
S5	BoNT/A	Meat extract			65/70							277	209	2	243	108	135	**4.9**	1.25	125
S6	BoNT/B	0.1% BSA/PBS			>180					87/88		10.0	9.0	2	9.5	9.0	0.5	**0.2**	0.06	5.7
S7	BoNT/A	0.1% BSA/PBS			65/60							277	376	2	327	100	227	**8.9**	2.27	227
S8A	BoNT/A	0.1% BSA/PBS				138		83/79				22.0 ^# §^	26.3 ^# §^	2	24.2	4.7	19	**16**	4.11	411
												11.4 * ^§^	8.7 * ^§^	2	10.0	4.7	5.3			
S8B	BoNT/B	0.1% BSA/PBS										5.60 ^§^	4.30 ^§^	2	4.95	4.5	0.4	**0.4**	0.1	10
S9	BoNT/A	0.1% BSA/PBS			>180					121/120		1.08	1.04	2	1.06	0.5	0.6	**4.5**	1.14	114
S10	BoNT/A	Milk			139		69/85					44.0	19.8	2	31.9	10.3	22	**8.2**	2.09	209
S11	BoNT/A	Serum			121		74/81					22.6	24.0	2	23.3	9.8	13	**5.4**	1.37	137
S12	BoNT/A	0.1% BSA/PBS	66/64	36	35							2.617 ^#^	2.942 ^#^	2	2.75	1001	−998	**−3.9**	−1	−100
												2617 *	2942 *	2	2780	1001	1779			
S13	BoNT/A	Milk			59/54							401	562	2	482	112	370	**13**	3.31	331

X_1,2_: reported concentration based on single measurement; X: mean of reported participant’s results; X_a_: nominal concentrations as determined by the organizing laboratory using sandwich ELISAs [54]; D: difference between X and X_a_; *z*: *z*-score $z=\;\frac{X-X_{a}}{\sigma }$; *Q*: *Q*-score $Q\left(\text{\%}\right)=100\left(\%\right)\cdot \frac{X-\;X_{a}}{Xa}$; * correctly calculated X_1_ and X_2_ based on paralysis times → ^#^ reporting error; § paralysis times denoted in Table 2 were used for calculation of X_1,2_.

**Table 2:** Identification of BoNT serotypes by neutralization with antiserum. 1:100 diluted samples (except S12 1:1000) were preincubated first with 1:400 diluted anti-BoNT/A antiserum (A). In cases where paralysis time (*t*_1/2_) exceeded 180 min, the BoNT serotype was supposed to be neutralized and assigned BoNT/A. In S8, anti-BoNT/A antiserum led to a partial neutralization (~80 min *vs.* ~130 min), thus beside BoNT/A another serotype had to be present. 1:400 diluted anti-BoNT/B antiserum only neutralized S6 (B) and 1:400 diluted anti-BoNT/E antiserum exclusively neutralized S4 (E). Neither anti-BoNT/B nor anti-BoNT/E antiserum neutralized S8. However, combined anti-BoNT/A and anti-BoNT/B antiserum completely neutralized the agents in S8. Paralysis times used for quantification of BoNT/A and BoNT/B in S8 are in bold and grey fields, respectively.

Anti-BoNT Antiserum	A	B	E	A + B	A + E	B + E	BoNT Serotype	
Sample #	Paralysis Time t_1/2_ (min)						Reported	Assigned
S1	>180						A	A
S2	>180						A	A
S4	85	74	>180				E	E
S5	>180						A	A
S6	109/103	>180	91				B	B
S7	>180						A	A
S8	134/121	**82**	80	>180	116	**88**	A + B	A + B
S9	>180						A	A
S10	>180						A	A
S11	>180						A	A
S12	>180						A	A
S13	>180						A	A

**Figure 2:**
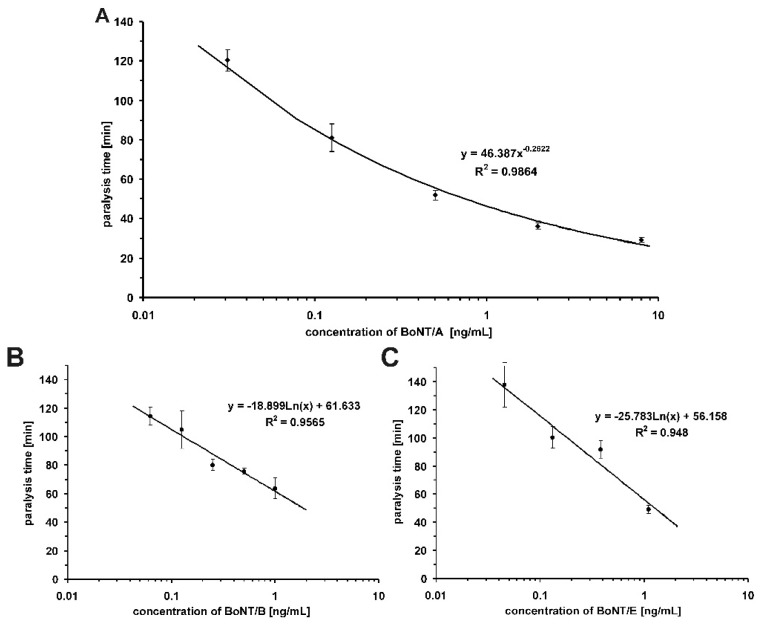
Calibration curves of BoNT/A (**A**); BoNT/B (**B**) and BoNT/E (**C**), respectively. The bath concentration of the respective BoNT is plotted against the paralysis time. Each data point is the mean of five (BoNT/A) and three (BoNT/B and E) single measurements ±standard deviation, respectively. To these calibration curves, depending on the orders of magnitude of BoNT concentration covered, power or logarithmic functions were fitted and used to quantify the various types of BoNT detected in the samples.

### 2.2. Experimental Procedures

The left phrenic nerve is nicely exposed soon after opening the chest, in contrast to the right phrenic nerve located far behind vital organs and closely attached to main blood vessels as well as connective tissue including fat. To obtain high rates of successful dissection only the left phrenic nerve hemidiaphragms were excised from male mice of strain RjHan:NMRI (18–22 g, St Berthevin Cedex, Janvier, France) and placed in an organ bath [11,12] containing 4 mL of EBSS supplemented with bovine albumin (0.1%). The pH was adjusted to 7.4 and oxygen saturation was achieved by gassing with 95% O_2_ and 5% CO_2_. The phrenic nerve was continuously electro-stimulated at a frequency of 1 Hz via two ring electrodes. The pulse duration was 0.1 ms and the amperage 25 mA to achieve maximal contraction amplitudes. Isometric contractions were recorded with a force transducer (Scaime, Annemasse, France) and the software VitroDat (FMI). The resting tension of the diaphragm was approximately 10 mN. Indirectly stimulated control muscles maintained an undiminished contractile response (twitch >65%) over 4 h (Figure 1). In each experiment, the preparation was first allowed to equilibrate for 15 min under control conditions. Then, the incubation solution was replaced by the toxin-containing solution (BoNT/A, B or E). After toxin application, the amplitude remained unchanged for some time (Figure 1), then decreased gradually depending on the toxin concentration. Toxin concentrations were such as to allow reduction of the contraction amplitude by 50% between 40 and 150 min. The times required to decrease the amplitude by 50% (paralysis time *t*_1/2_ ≤ 180 min) at different BoNT concentrations were used to construct the calibration curves for BoNT/A, B and E (Figure 2). Optimal power or logarithmic functions depending on the spread of the calibration curves were fitted to the calibration curves: *y* (BoNT/A; 0.031, 0.125, 0.5, 2, 8 pg/mL) = 46.387*x*^−0.2622^, *R*^2^ = 0.9864, *y* (BoNT/B; 0.0625, 0.125, 0.25, 0.5, 1 ng/mL) = −18.899Ln(*x*) + 61.633, *R*^2^ = 0.9565, *y* (BoNT/E; 0.378, 0.131, 0.045, 1.098 ng/mL) = −25.783Ln(*x*) + 56.158, *R*^2^ = 0.948. Resulting paralytic half-times of the diluted samples were converted to BoNT concentrations in the 12 samples employing the above fitted functions.

## 3. Results

Because only 1 mL of each sample was available, in a first series of experiments each of the 13 samples (S1–S13) delivered [54] was diluted in EBSS by a factor of 1000 to save material and tested for BoNT activity. If paralysis occurred and paralysis time was below 120 min, the sample was judged positive for presence of substances that cause paralysis of a nerve muscle preparations. When paralysis time was below 55 min the sample was further diluted until paralysis time was within the range of the calibration curves, *i.e.*, >50 min. If the paralysis time lay outside their ranges the original sample was diluted again with a lower factor (Table 1). This procedure was repeated until a factor of 40 was reached. To avoid effect of matrices, samples to be diluted 1:40 or less were dialyzed against EBSS before being added into the organ bath. Dialysis did not affect activity of toxins. Table 1 demonstrates that paralysis times of all tissue preparations except of that treated with S3 were within the range of the calibration curves and the respective samples were therefore judged positive *i.e.*, they were contaminated with a substance that causes paralysis of a nerve muscle preparation. These numbers were used to calculate the concentration of BoNT within the bath solution. Sample 3 was diluted by a factor of 13 and the test was repeated. Again, the paralysis time exceeded the range of the calibration curves.

The results demonstrate that 12 out of 13 samples contained substances leading to muscle paralysis. In a second series of experiments, the cause of paralysis was examined with the help of specific polyclonal antibodies against BoNT/A, B and E, respectively. All positive samples were tested again after the 4 mL sample containing solution was incubated for 60 min with 10 µL rabbit anti-BoNT/A antiserum (bath concentration 0.75 U/mL) at 37 °C. In nine out of 12 samples, the paralysis time exceeded 180 min, thus the muscle paralyzing substance was neutralized (Table 2). Hence, it was concluded that these matrices were contaminated with BoNT/A. This procedure was repeated with the remaining three samples, however, instead of anti-BoNT/A, 10 µL of anti-BoNT/B (1.25 U/mL) and anti-BoNT/E (0.25 U/mL), respectively, were tested for BoNT neutralizing activity. As shown in Table 2, S6 could be neutralized by anti-BoNT/B and S4 by anti-BoNT/E, consequently S4 contained BoNT/E and S6 BoNT/B, respectively. However, the tissue paralyzing substance in S8 was not neutralized by any anti-BoNT, but anti-BoNT/A prolonged paralysis time considerably, leading to the suspicion that besides BoNT/A at least a second tissue paralyzing substance was present. Therefore, in the final series of experiments dual combinations of the three anti-BoNT antisera were tested for neutralizing activity. Only the combination of anti-BoNT/A and anti-BoNT/B could neutralize the paralyzing capability completely. Thus, the paralysis time obtained in the presence of anti-BoNT/A represents the time due to BoNT/B, and the paralysis time achieved when anti-BoNT/B was present represents the time due to BoNT/A (Table 2).

With the help of the calibration curves, the concentrations of the respective BoNT were calculated and these concentrations were then multiplied by the dilution factor. These results were compared with the nominal values (see Table 1).

## 4. Discussion

The potential misuse of BoNTs as biological weapons or terrorist tools [56], the occurrence of accidental intoxications caused by consumption of contaminated food [53] and the usage of BoNT as a therapeutic drug [57,58] require sensitive methods for the detection of minute amounts. Because of their extremely high potency, the concentrations of clinically effective or even deadly BoNT doses are below the limit of detection of common chemical detection systems. It is, therefore, of utmost importance to develop reliable methods that can be used to economically screen food, water or air for possible toxin contaminations in the case of potential terrorist attacks, to analyze body fluids and stool in case of potential intoxication and to measure the potency of therapeutic products for safety reasons. The demands of the various methods differ with respect to the different purposes. In some cases it might suffice to detect simply the presence of the protein in its active or inactive form [1]. However, immunological methods are especially error-prone in terms of false negative results due to the vast diversity of BoNT [59] and the occurrence of novel variants [60]. Functional methods will only detect active, and thus dangerous, BoNT independent of its primary structure. For BoNTs manufactured for therapeutic purposes, non-functional methods are not appropriate to prove activity, stability and quality of the drug, because all steps leading to blockage of vesicle fusion in nerve cells are essential for activity and have to be covered by the test. Therefore, a method used for quality control must test the function of each domain of the neurotoxin (binding, translocation, release of enzymatic domain and its catalytic activity) in one assay or in a set of assays. If a single assay is desired, this can only be performed by *in vivo*, *ex vivo* and cellular assays. The MBA still represents the most customarily employed method for detection of BoNT in complex matrices, as well as its precise quantification for pharmaceutical purposes, and has been accepted by regulatory agencies worldwide [9,17]. However, its major and multiple disadvantages have been described above and elsewhere [9,61]. Since the endpoint of the test is the paralysis of the respiratory muscle, a truncated version of the test is represented by an isolated nerve-muscle, the phrenic nerve hemidiaphragm preparation [11,12,13], which has been successfully employed by several laboratories worldwide to determine potency of BoNT preparations, decipher basic science mechanisms, identify BoNT inhibitors, characterize BoNT-neutralizing antibodies, and screen sera of BoNT-treated patients for development of BoNT neutralizing antibodies. Its usefulness in detecting BoNT in complex matrices has only been challenged in rare botulism cases so far [53].

Within the present EQuATox BoNT proficiency test, 13 blinded samples had to be analyzed with respect to presence or absence of BoNT/A, B, E and/or F and, if possible, also with respect to quantity of BoNT. Since the MPN assay detects only active BoNT, inactivated or denatured toxin escapes the detection. Furthermore, muscle paralysis caused by BoNT-derived activity has to be delimited against any other muscle-paralyzing substance. On the one hand, the time course of paralysis is very characteristic for BoNT. In contrast to peripheral muscle relaxants or channel blockers that act promptly after application, paralysis due to BoNT starts after a concentration dependent latent period of >25 min and shows a slow and progressive decline of contractions. This characteristic course may be mimicked by slow-acting cellular poisons like ricin [62]. However, on the other hand, BoNT activity can be neutralized by highly serotype-specific anti-BoNT antibodies. Thus, with the help of monovalent antiserum it should be possible to identify a BoNT serotype. For this proficiency test anti-BoNT/A, B and E antisera were available; consequently, other serotypes would have evaded detection.

The analysis of all 13 samples was performed with 63 individual MPN tests, of which 40 tests were run to quantify the amount of toxin and 23 tests to determine the serotype. Here, only the left hemidiaphragm was dissected, so, including wastage, only 66 mice were consumed. Dissecting both halves of the diaphragm would have even halved the number of mice required. Beside the absence of any animal suffering with the low amount of mice consumed, the MPN assay clearly outnumbers the MBA, which required more than 500 mice in some laboratories for a semi-quantitative analysis of the PT samples. Although this time, the PT sample analyses were performed randomly over a period of three weeks, in an emergency situation considering the technical capacity of toxogen, the same analysis, including sample preparation (dialysis), serotype identification and quantification, would have been finished within 2–3 days whereas one run of the MBA already requires 4 days.

Out of 13 samples only one sample (S3) was free of paralyzing activity and thereby presumably free of any BoNT. However, with respect to the limit of detection, it cannot be excluded that this sample might contain a BoNT at a concentration of less than 30 pg/mL in the cases of BoNT/A and 50 pg/mL of BoNT/B and E, respectively (Figure 2). All remaining samples paralyzed the muscle. BoNT/A alone was identified in 9 samples, a combination of BoNT/A and B in S8 and only BoNT/B and BoNT/E in S6 and S4, respectively. The sample matrices did not impair the analysis at any point. In summary, all 13 samples were correctly analyzed (Table 2) by the MPN assay, which had a performance superior to several immunological assays and some MBAs performed in other participating laboratories [54]. 100% qualitatively correct results were also returned by 4 out of 5 Endopep-MS assays [63], two sandwich-ELISAs, a single LFA and 4 out of 9 MBA [54].

With respect to the quantification of BoNT/A, a mean *z*-score of 7 ± 5.7 and a mean *Q*-score of 1.79 were achieved. The true mean *Q*-score excluding a miscalculation (S8A) and a reporting error (S12: language specific punctuation translation error) is +1.85. This two-fold higher quantification of BoNT/A reflects the fact that the BoNT/A calibration curve was established with a BoNT/A from an external supplier in the absence of certified reference material. On the other hand, quantification of BoNT/B and E yielded *z*-scores of 0.3 and 1.6, respectively (Table 1). Here, the calibration curves were generated employing the same reference material [55] used for spiking PT samples S4, S6 and S8 [54], which strongly supports the urgent provision of internationally available certified BoNT reference material. Furthermore, it demonstrates the high precision of the MPN assay.

In conclusion, the analysis of the EQuATox PT samples demonstrated the power of the MPN assay in detecting functionally active BoNT in complex matrices in a short time with high precision in a laboratory environment, but relies on availability of appropriate anti-BoNT antisera for serotype identification and qualified reference material for precise quantitation.
